# Effect of resistance and endurance training with ursolic acid on oxidative stress and cognitive impairment in hippocampal tissue in HFD/STZ-induced aged diabetic rats

**DOI:** 10.22038/IJBMS.2023.71230.15477

**Published:** 2023

**Authors:** Safoura Alizade, Mohammad Faramarzi, Ebrahim Banitalebi, Elham Saghaei

**Affiliations:** 1 Department of Sport Sciences, Shahrekord University, Shahrekord, Iran; 2 Department of Exercise Physiology, Faculty of Sport Sciences, University of Isfahan, Isfahan, Iran; 3 Department of Pharmacology, Shahrekord University of Medical Sciences, Shahrekord, Iran

**Keywords:** Cognitive impairment, Diabetes, Hippocampus, Nrf2/Keap1/ARE signaling, Training, Ursolic acid

## Abstract

**Objective(s):**

The increase in age-related cognitive impairment (CIs) and diabetes mellitus is a global health concern. Exercise training has been reported to activate the Nrf2/Keap1/ARE signaling and enhance the antioxidant defense pathways in some animal models. This study aimed to investigate the effects of ursolic acid (UA) associated with resistance or endurance training on antioxidant markers, and the Nrf2/Keap1/ARE pathway in the brain of older diabetic rats.

**Materials and Methods:**

23-month-aged diabetes induced male Wistar rats were randomly assigned to seven groups (n=8). UA supplementation (250 mg/kg, daily) was administered along with resistance (60% maximum capacity of voluntary carrying [MVCC], 14-20 climbs) or endurance training (60-75% velocity at maximal oxygen uptake [vVO_2max_]), five days/week for eight weeks. Cognitive-motor functioning was assessed through open-field and passive avoidance response tests. Nrf2, Keap1, and antioxidant markers including SOD, CAT, GPx, and GSH were measured in the hippocampus tissue.

**Results:**

The results showed positive effect of resistance training (*P≤*0.001) on Nrf2. There was endurance training with supplementation main effect (*P=*0.018) on Keap1 concentration. SOD revealed a significant endurance/resistance training by supplementation interaction effect (*P≤*0.05); however, there was no main training or UA supplementation effects on CAT, GPx, and GSH, despite improving spatial memory changes in exercise or UA groups.

**Conclusion:**

It appears that UA treatment with resistance or endurance exercise has some beneficial effects on Nrf2 and some antioxidant markers. However, more research is needed to elucidate UA’s interaction effects and exercise interventions in diabetic situations.

## Introduction

Type 2 diabetes mellitus (T2DM) is known as a common metabolic disorder characterized by hyperglycemia and increased insulin resistance (IR)(1). Insulin signaling and normal glucose metabolism are vital for normal brain function. Intravenous administration of either insulin while maintaining normal blood glucose levels or glucose alone facilitates cognitive functions in patients with AD and healthy older adults (2). Thus, normal glucose metabolism is required to perform cognitive functions, and data suggest that impairments in glucose metabolism may contribute to cognitive dysfunction (3). 

A common theory for aging and the pathogenesis of this cerebral dysfunction in diabetes relates cell death to oxidative stress-mediated free radicals. Such effects contribute to tissue damage in diabetes, giving rise to alterations in the redox potential of cells with the subsequent activation of redox-sensitive genes (4). According to some studies, diabetes can have adverse effects on cognitive-motor functioning, like memory and learning, but the mechanism of diabetes-associated cognitive impairments (CIs) is not entirely clear (5, 6). CIs also occur in rats with high-fat diet-streptozotocin (HFD-STZ)-induced diabetes. 

The nuclear factor erythroid 2-related factor 2 (Nrf2) transcription factor is coded by the NFE2L2 gene, a member of the family of bZIP transcription factors (basic Leucine ZIP per), is known as the anti-oxidant cytoprotection master regulator, exerting an impact on more than 200 genes associated with the oxidative stress (OS) response (7). Under normal cellular conditions, Nrf2 remains inactive by binding its negative regulator Keap1 (Kelch-like ECH-associated protein 1), a cysteine-rich protein that the presence of ROS can oxidize and, as a consequence, it changes its conformational state, releasing Nrf2 (8). Thus, the increase of OS promotes the dissociation of Nrf2 from Keap1, giving rise to its translocation in the nucleus (9), where it binds to the anti-oxidant response element (ARE), activating a large group of genes associated with the anti-oxidant and detoxifying response, such as superoxide dismutase (SOD), glutathione peroxidase (GPX), glutathione (GSH), and catalase (CAT), as well as detoxification genes (10, 11). Accordingly, research has shown that Nrf2/Keap1/ARE signaling pathway may be associated with IR in the brain and CIs (12). The high expression of Nrf2 can also resist oxidative stress and improve insulin signaling function in the hippocampus *in vitro* (12) .

Exercise is a critical instrument in managing chronic conditions associated with inflammation and oxidative stress and may be an effective method to promote a cellular protective effect through endogenous anti-oxidants. In response to the increase of ROS during physical training, several crucial endogenous anti-oxidant enzymes regulate redox homeostasis, such as Nrf2, catalase (Cat), superoxide dismutase (SOD), and glutathione peroxidase (GPx)(13). Nrf2 signaling pathways are important parts of the exercise-induced adaptive response to oxidative challenge. Since neuronal mitochondria are one of the prominent sites of ROS generation during exercise, the Nrf2-mediated adaptive response could play an important role in producing the beneficial effects of exercise (14). As the intensity or duration of the exercise increases, the metabolism of hippocampus and hypothalamus increases, resulting in necessary up-regulation of the Nrf2 pathway and anti-oxidant capacity (15). In this regard, several studies have further examined the effects of different exercise training modes on some oxidative stress biomarkers in different people (16, 17). However, there is no consensus about Nrf2 response and adaptation to various types of exercise training. To the best of the authors’ knowledge, few studies have been reported on Nrf2 changes in the brain in response to different types of exercise training.

Along with exercise training interventions, the prescription of some herbal supplements for greater effectiveness has been even considered by researchers. In this sense, ursolic acid (UA) is a natural pentacyclic triterpenoid carboxylic acid extracted from the leaves of various plants (such as apple peel) (18). Studies in this regard have shown that UA supplementation can regulate blood glucose levels and improve memory dysfunction induced by HFD-STZ (19, 20). It has been shown that UA can enhance the neuroprotective effects by increasing the expression of Nrf2 (21). Ding and colleagues revealed that UA boosted the expression of AKT, an Nrf2 upstream gene, and sensitizes the Nrf2 pathway in protecting against cerebrum damage (22). In contrast, UA expresses no neuroprotective influence on Nrf2‐deficient mice. It has been revealed that UA can enhance the activation of antioxidative enzymes and lessen cerebrum damage through the Nrf2 signal pathway (22). Therefore, it has been proposed that UA can activate the Nrf2 signaling pathway, and it has become a potential therapeutic target for reducing oxidative stress associated with chronic age-related neural disfunctions. However, to our knowledge, there is limited research on combined exercise training and anti-oxidant intervention on the Nrf2/Keap1/ARE signaling pathway. The interaction between exercise training and anti-oxidant supplementation has not been further evaluated thoroughly; therefore, this study was to determine the rate of changes in the Nrf2/Keap1/ARE signaling pathway and SOD, CAT, GPx, GSH anti-oxidant markers along with endurance/resistance exercise training with/without UA supplementation in the hippocampal tissues of the brain of aged diabetic rats.

## Materials and Methods 

***Ethical approval***

The Veterinary Ethics Committee approved the study and animal care procedures based on the Animal Research and Ethics at Shahrekord University, Shahrekord, Iran (IR.SKU.REC.1399.001). All the experiments were conducted in compliance with the National Institute of Health Guide for the Care and Use of Laboratory Animals.

***Animal handling and group assigning***

**Figure 1:**
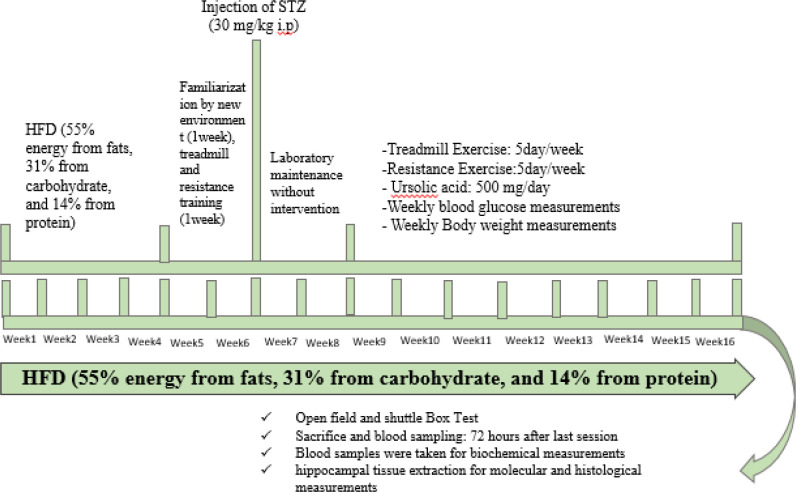
A summary of the experimental design

The animals recruited in this study were 56 experimental 21-month-old male Wistar rats (427±44 g) obtained from Pasteur Institute of Iran, Tehran, Iran. The animals were accordingly housed in an air-conditioned room at 20±2 ^°^C with a 12-hr:12-hr light/dark cycle (light 08:00 a.m. -08:00 p.m.) and humidity (40±65%). They were also housed at 2-3 cases per cage. In total, 56 rats were used for this experiment, which were randomly selected and divided into seven groups (n=8), (1) sedentary aged non-diabetic (C) group fed a standard rodent-chow diet, (2) sedentary HFD/STZ-induced Diabetes (D) group fed HFD/low-dose STZ, (3) sedentary HFD/STZ-induced Diabetes plus UA (DU) group fed HFD/low-dose STZ plus UA (250 mg UA per kg of body weight rat/day), (4) endurance-trained HFD/STZ-induced Diabetes (DE) group fed HFD/low-dose STZ plus endurance exercise training performed at 60-75% vVO_2max_ for five days/week, (5) resistance-trained HFD/STZ-induced Diabetes (DR) group fed HFD/low-dose STZ plus resistance exercise training performed at 60% MVCC, 14-20 climbs, five days/week, (6) endurance-trained HFD/STZ-induced Diabetes plus UA (DEU) group fed HFD/low-dose STZ plus UA (250 mg UA per kg of body weight rat/day) plus endurance exercise training performed at 60-75% vVO_2max_ for five days/week, and (7) resistance-trained STZ-diabetic plus UA (DRU) group fed HFD/low-dose STZ plus UA (250 mg UA per kg of body weight rat/day) plus resistance exercise training performed at 60% MVCC, 14-20 climbs, five days/week (Figure 1).

***HFD/STZ-induced diabetes mellitus in rats***

Diabetes mellitus was induced using low-dose STZ purchased from Sigma-Aldrich Corp. (Aldrich, St Louis, MO, USA). All the rats except the control group were then fed for four weeks with an HFD formulated with energy from fat (55%), carbohydrate (31%), as well as protein (14%)=5.2 kcal/g. However, the control group rats were only fed a regular basal diet (10% fat, 75% carbohydrate, and 15% protein, Royan Co., Isfahan. Iran). After four weeks, STZ (30 mg/kg dissolved in 0.1 mol/l sodium-citrate buffer at a pH of 4.4-4.5) was IP injected (23). The control group rats were injected with an equivalent volume of citrate buffer (as a vehicle) (0.25 ml/kg) alone. After 72 hr, the fasting blood glucose level was measured, so the animals with a blood glucose concentration ≥200 mg/dl were considered diabetic and thus chosen for additional investigations. The blood glucose levels were tested using the glucometer after the first, second, fourth, sixth, and eighth weeks (bionime, GM110, Switzerland). UA (250 mg UA per kg of body weight rat/day) was also prepared by mixing 500 mg of UA per kg of an HFD (0.5% UA plus HFD, Royan Co., Isfahan. Iran)(24). The average daily amount of food received was further calculated as the difference between leftover food and the whole food provided, divided by the number of days and rats in the cages. As the amount of energy varied between the diets, the use of food in grams was converted into calories (25). Finally, body weight was measured every week for each rat during the study (26). 

***Endurance training protocol***

At first, the rats were adapted to the treadmill (Danesh Salar Iranian Company, Iran). Then, vVO_2max_ was measured according to the standard incremental test developed by Leandro *et al*. (2007). The test consisted of ten 3-min phases with zero slopes and 0.3 km/hr speed in the first phase and addition of 0.3 km/hr to the treadmill’s speed in the next phase. In the last phase, the maximum running speed was also considered when the rats could not run (27). The endurance training protocol was performed at zero slopes, five days/week for eight weeks. Then, in the fourth and eighth weeks, the vVO_2max_ was determined to monitor the ability of the aged diabetic rats to perform the endurance training protocol. The moderate-intensity interval training (MIIT) program also included a five-min warm-up running on the treadmill at 40-50% vVO_2max_, running at 60-75% vVO_2max_, and a five-min cool-down by running at 40-50% vVO_2max_. The MIIT protocol also contained two minutes running with 60% vVO_2max_ on the first week, 65% vVO_2max_ during the second week, 70% vVO_2max_ within the third week, and finally 75% vVO_2max_ in the fourth week to the completion of the exercise training time. Besides, the low-intensity training involved two-min running with 40% vVO2max from the first week to the end of the third week and 30% vVO2max from the fourth week to the completion of the eighth week (28).

***Resistance training protocol***

All rats were adapted to the climbing apparatus for one week, which included a vertical ladder (110 cm, 2-cm grid, 85^o^ inclines) without overloading and a tail weight attachment. To determine the maximum capacity of voluntary carrying (MVCC), 75% of their body weight, the weight was attached to the tail, and the animals began to climb the ladder by carrying this load. At the top of the ladder, they were further allowed to rest for two min between each climb. Then, a 30-g weight was added for each successful repetition until total fatigue was reached. To climb the entire length of the ladder on three consecutive attempts, the MVCC was then defined as the most significant load successfully carried. The MVCC was accordingly measured at the beginning and after the fourth and eighth weeks of training (29). The resistance training protocol included eight weeks of ladder resistance training at 60% MVCC and 14 or 20 climbs per session with a one-min rest between each trial, five days per week (30). 

***Open-field test***

The open-field test was performed to assess spatial memory. Getting used to the open field consisted of placing an animal in the open field in a new environment without unpleasant or pleasant stimuli. The animals were then allowed to explore the area freely for a specified time. The desired time was five minutes. A square wooden box 50×60×40 cm in size was thus used. The floor of the open field was also divided into equal parts by white lines into 16 equal squares of 4×4. At first, each rat was placed gently in the central area and allowed to explore it freely for five min. After testing each rat, the floor and the walls were cleaned to eliminate possible bias due to the smells left by previous rats. The items measured for each rat included the time of placement in the central area, the number of times the animal stood and stretched on its hind limbs with/without forelimb support in the mentioned time, and the number of squares crossed by the animal within the five min designated.

***Passive avoidance response test***

The shuttle box, used to assess passive avoidance behavior while passing, consisted of a two-chamber dark/light shuttle box (35×35×35 cm) with a rectangular opening (15×15 cm) located between the two chambers. One of these two compartments was the light compartment (viz. safe area) which had white walls, and the other was the dark box (namely, unsafe area), whose walls were black. Intermittent electric shock (100 V, 0.3 mA, and 0.5 sec) rods also covered the floor of the dark chamber and were connected by wires to the electroshock device. Passive avoidance behavior was then assessed for two consecutive days. The first day of the session was exercise training, which had two phases, namely, the automation session wherein all the rats were placed in the laboratory for at least 30 min before the start of the experiment. Then, each rat was placed gently in a light chamber, then, the guillotine door was opened after five seconds, and the animal was allowed to enter the dark room. The initial delay was noted as the time it took for the animal to enter the dark chamber from the lightroom. The criterion for the animal to enter the dark room was the entry of the hind legs into the room, and at this time, it immediately closed the guillotine door, and an electric shock was transmitted. After 10 sec, the animal was returned to its cage. The next day, the rat was placed in the light room again, and the guillotine door was opened. The guillotine door was closed, and the animal was shocked and returned to the cage as soon as it entered the dark room. Two minutes later, the animal was placed in the light room again, and if it re-entered the dark room, it was shocked again, but if it had learned and did not enter the dark room for two minutes, the test ended.

***Brain processing***

After the behavioral testing, the rats were weighed and anesthetized through IP administration of a mixture of 90 mg/kg ketamine and 10 mg/kg xylazine through the left ventricle to remove blood from the vessels. Then, their hippocampus was quickly collected on ice and stored at -80 ^°^C for further analysis. 

***Western blotting analysis***

Western blotting was performed on the hippocampal tissue homogenates. In summary, the lysis buffer (consisting of protease inhibitor cocktail, Tris-HCL, Triton NP40 [1%], EDTA, NaCl, Sodium Deoxycholate, and SDS) was used to lubricate the tissues, and then the samples were centrifuged in a centrifuge (Eppendorf 5415 R) at 4 ^°^C at 12,000 rpm for 10 min. Subsequently, the clear liquid containing the protein was extracted and stored in a -20 ^°^C freezer. The Bradford protein assay (Bio-Rad Laboratories, CA, USA) was employed to determine protein concentration. Bovine serum albumin (BSA) was also used as a standard protein to measure protein levels. Membranes were blocked with 5% BSA in TBST, followed by incubation with specific primary antibodies (Nrf2, Keap1, as well as β-actin (Santa Cruz, USA) 1:300) overnight at 4 ^°^C, and then incubated with corresponding HRP-conjugated, anti-rabbit/mouse secondary antibodies (mouse anti-rabbit (IgG) (Santa Cruz, USA) 1:1000) for 1 hr at room temperature. Finally, antiβ-actin was employed as one of the loading controls. The band intensity on immunoblots was further quantified via densitometry using the Image Studio Lite software.

***GSH assay***

The GSH level was determined using Ellman’s reagent (5,5′-dithiobis-[2-nitrobenzoic acid] or DTNB) as an indicator. In brief, a sample (0.5 mg protein/ml) was added to DTNB (0.04%), and a spectrophotometer was employed to read the produced yellow color at 412 nm (UV-1650PC, Shimadzu, Japan).

***CAT assay***

The ZellBio GmbH CAT activity assay kit was also utilized to determine CAT in biological samples with 0.5U/ml sensitivity (0.5KU/L). In this assay, the CAT activity unit was considered as the amount of the sample that could catalyze the decomposition of 1 µmol of hydrogen peroxide (H_2_O_2_) to water (H_2_O) and oxygen (O_2_) in one min.

***SOD assay***

The ZellBio GmbH SOD activity assay kit was similarly employed to find SOD activity in biological samples with 1 U/ml sensitivity (1 KU/L). In this assay, the SOD activity unit was considered the sample amount that could catalyze the decomposition of 1 µmol of O_2 _to H_2_O_2_ and O_2_ in one min.

***GPx assay***

The ZellBio GmbH GPX activity assay kit was further used to determine GPx in biological samples with 5U/ml sensitivity (5KU/L). In this assay, the GPx activity unit was considered the sample amount that could catalyze the decomposition of 1 μmol of GSH to oxidized glutathione (GSSG) in one minute.

***Data analysis***

Statistical analyses were performed using the SPSS Statistical software (ver. 21) and figures generated using GraphPad Prism statistical software (version 8. 4; GraphPad Software Inc., San Diego, CA, USA). Data from Nrf2, Keap1, SOD, GP_X_, CAT, and GSH concentration and behavioral tests were analyzed by one-way ANOVA but Blood Glucose and Weight Changes, MVCC and vVO_2max_ data were analyzed by repeated measurements, respectively, and followed by Tukey’s *post hoc* test. Within-group effects were calculated by using paired-sample t tests. The results of this research are presented as the Mean±SEM and reflected at *P* values less than 0.05.

## Results

***Blood glucose and weight changes in rats in each group***

**Figure 2:**
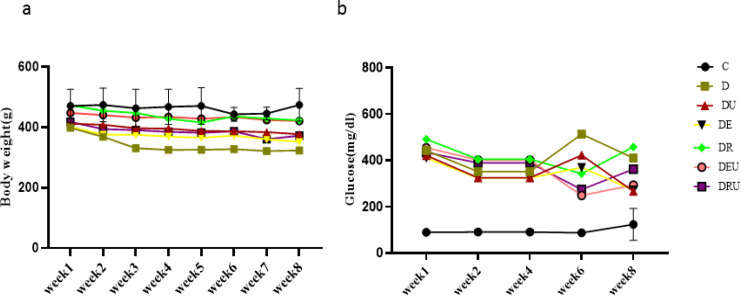
Bodyweight (a) and changes in blood glucose levels (b) following eight-week exercise training and UA supplementation

To determine the effect of STZ-induced diabetes on body weight these variables were measured intermittently. Body weight changes following the eight-week interventions was not significant in time*groups (*P*=0.65) but a considerable time main effect (*P*=0.001). BW decreased in the D group compared with C (*P*=0.019). Moreover, Within-group effects demonstrated that body weight had been significantly boosted in the DRU (*P*=0.035), DR (*P*=0.021), DE (*P*=0.042), and DU (*P*=0.046) groups in the eight weeks compared with the first week (Figure 2a).

Also, Glucose concentration showed a significant main time effect (*P*=0.013) and time*group main effect (*P*=0.037). The *post hoc* comparisons for the group main effect also revealed that glucose levels had significantly increased in the DEU, DE, DR, DU, D, and DRU groups compared with the C one (*P*≤0.001). Further more, within-group effects correspondingly established that glucose levels had remarkably elevated in the DU (*P*=0.005) group in the eight week compared with the first week (Figure 2b).

***MVCC and vVO***_2max_ ***changes in study groups***

**Figure 3:**
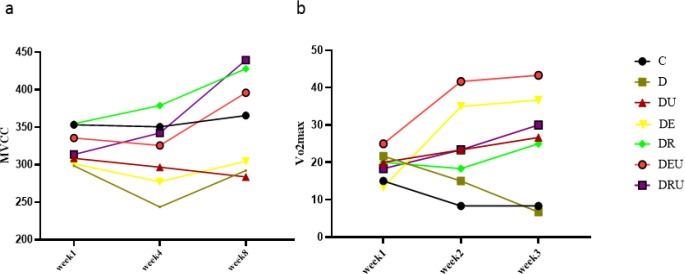
MVCC (a) and vVo_2max_ (b) following eight-week training and UA supplementation

A significant time main effect (*P*=0.001) and time*group main effect (*P*=0.016) was observed for MVCC. The *post hoc* comparisons for the group main effect also revealed that MVCC had remarkably enhanced in the DR groups compared with the D one (*P*=0.028). Moreover, within-group effects demonstrated that MVCC had been significantly boosted in the DRU (*P*=0.031) and DU (*P*=0.046) groups at the post-test stage compared with that at the pre-test (Figure 3a). 

As well, vVo_2max_ showed a significant main time effect (*P*=0.001) and time*group main effect (*P*=0.001). The *post hoc* comparisons for the group main effect also revealed that vVo_2max_ had significantly increased in the DEU, DE and DRU groups compared with the C one. This was similarly evident in the DEU and DE groups compared with the D one and the DEU group compared with the DRU and DU ones (*P*<0.05). Moreover, Within-group effects correspondingly established that vVo_2max _had remarkably elevated in the DEU (*P*=0.008), DE (*P*=0.005), DU (*P*=0.057), D (*P*=0.035), and C (*P*=0.057) groups in the eighth week compared with the first week (Figure 3b).

***Serum glucose and insulin changes in study groups***

**Figure 4:**
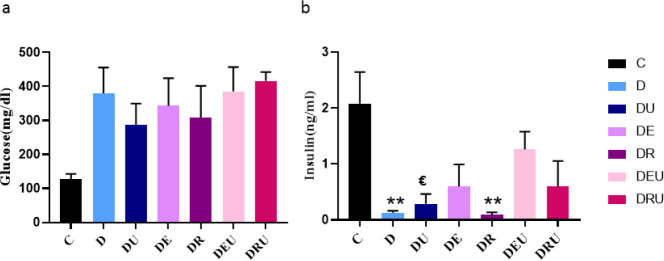
Serum Glucose (a) and insulin (b) following eight-week exercise training and UA supplementation

In the blood glucose concentration there was no difference in between groups throughout the study (F=2.126, *P*=0.115) (Figure 4a). 

Insulin signaling deficiency in the hippocampus is known to modulate cognitive function. As shown in (Figure 4, b), a significant difference was observed in insulin levels (F=4.360, *P*=0.011). Rats treated with resistance training or ursolic acid supplementation had significantly decreased expression of insulin (*P<*0.01).

***Expression of Nrf2/Keap1/ARE signaling pathway changes in study groups***

**Figure 5:**
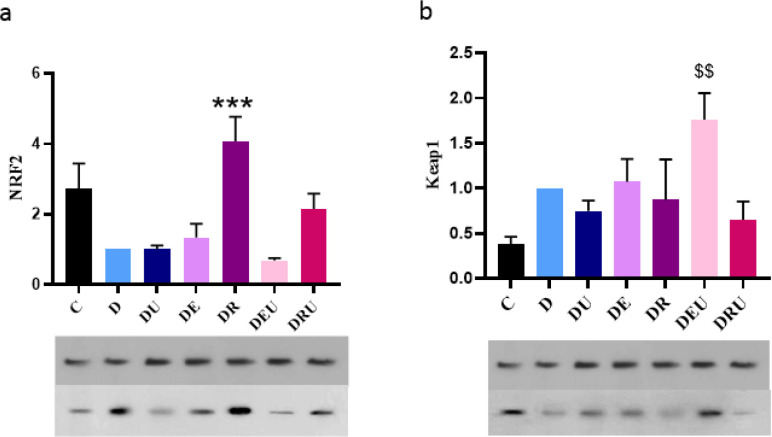
Expression of Nrf2/Keap1 signaling pathway changes (a, b)

By one-way ANOVA a significant difference was observed in the level of Nrf2 (F=7.438, *P*=0.001). The *post hoc* comparisons also demonstrated that Nrf2 levels had significantly increased in the DR groups compared with the DEU (*P*=0.001), DE (*P*=0.009), EU (*P*=0.004), and DC (*P*=0.004) (Figure 5a). 

As shown in (Figure 5b), a significant difference was observed in level of Keap1 (F=3.133, *P*=0.037) of different groups. training by supplementation interaction significantly increased hippocampal Keap1 level in the DEU group in comparison with the C group (*P<*0.018). 

***Expression of SOD, GP***_X_ ***, CAT, and GSH changes in study groups***

**Figure 6:**
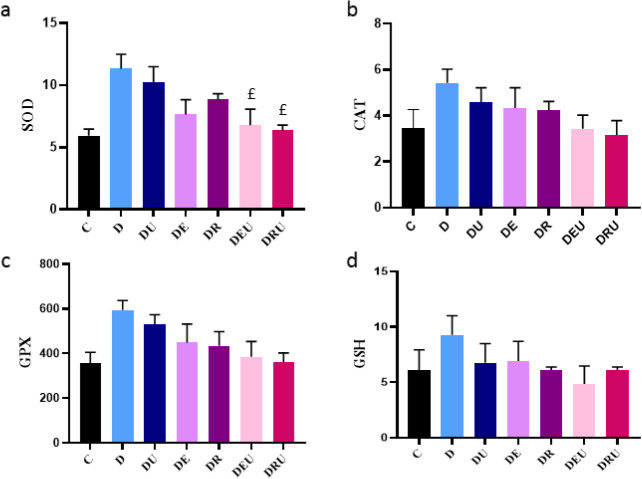
Expression of SOD, GPX, CAT, and GSH changes (a, b, c, d)

The one-way ANOVA results established a significant difference in level of SOD (F=4.346, *P*=0.011), so that rats treated with training by supplementation interaction significantly increased SOD activities in the hippocampus of DRU and DEU groups rats compared to the D group (*P<*0.05). (Figure 6a). Similarly, there was no significant difference in GPx (F=2.418, *P*=0.081), CAT (F=1.427, *P*=0.272), and GSH (F=0.685, *P*=0.666) activities observed between groups rats (*P>*0.05)(Figure 6b, c, d). 

***Open-field test***

**Figure 7:**
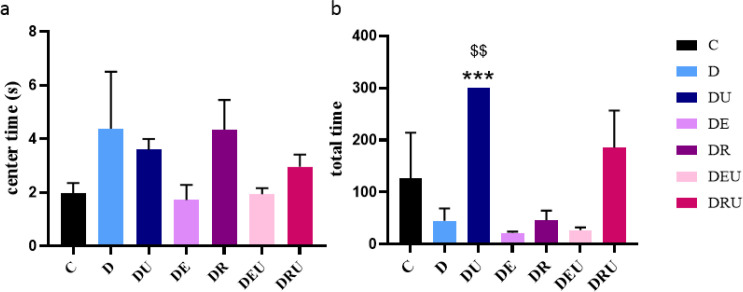
Open-field test (a, b)

Cognitive dysfunction is a consequence of diabetes, leading to memory and learning impairments. We examined the effects of diabetes on cognitive function with the open-field test in STZ-induced diabetic rats. As shown in (Figure 7), in the Open-Field test, significant differences in spatial memory of different groups were observed on the total time (F=5.562, *P*=0.004), so that STZ-induced diabetic rats in DU group showed significantly higher spatial memory in the total time compared to the DEU (*P=*0.009), DE (*P=*0.008) and DR (*P=*0.015) group rats (Figure 7b). Likewise, as shown in Figure 7a, a significant difference was not observed in the time spent in center time (F=1.393, *P*=0.284). 

***Passive avoidance response test***

**Figure 8:**
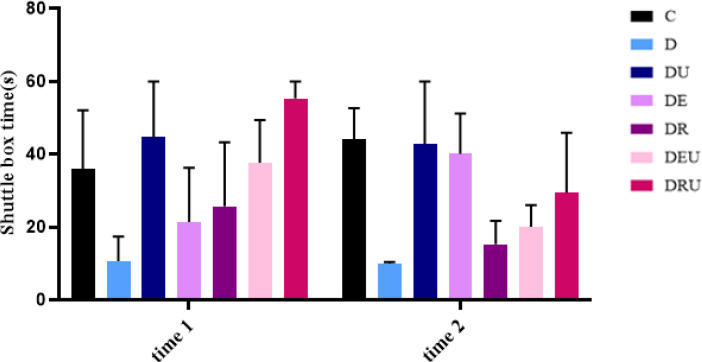
Passive avoidance response test

Following 8 weeks of the experiment, a significant difference in passive avoidance response test was not observed between the study groups (F=1.283, *P*=0.326) (Figure 8).

## Discussion

The present study aimed to examine the effects of resistance/endurance training with/without UA supplementation on the Nrf2/Keap1/ARE signaling pathway and explore the possible mechanisms underlying its therapeutic effects on insulin signaling, oxidative stress, and CIs in HFD/STZ-induced aged male Wistar rats with diabetes. In addition to investigating the effect of diabetes on hippocampal tissues and CIs, the therapeutic goals of resistance/endurance exercise training and UA supplementation were also evaluated alone and in combination.

Glucose dependence by the brain and its elevated levels of polyunsaturated fatty acids and exacerbated metal loads make it vulnerable to oxidative damage (31). Glucose metabolism disorder and IR in the hippocampus of diabetic rats could be thus closely related to CIs, dysfunctional glucose metabolism, and brain metabolic stress, which might damage cognitive-motor functioning, playing a role in the destruction of the insulin receptor system (32). Studies have reported the beneficial role of natural product anti-oxidants in treating and managing neurodegenerative diseases with plant phytochemicals majorly constituting these natural products (33). These roles stem from scavenging free radicals and modulating intrinsic anti-oxidant enzyme activities (33). Jang *et al*. (2009) showed that UA inhibits glucose intolerance and insulin resistance by preserving pancreatic β-cells in diabetic mice. Similarly, 0.5% UA-supplemented diet treatment at a dosage of 0.5 mg/kg had therapeutic effects on the insulin responsiveness and body weight of HFD/STZ-induced diabetic rats without affecting the anti-oxidant response (34). Here, our study results showed that UA with/without training did not change body weight, and no improvement in glucose metabolisms such as serum glucose and insulin concentrations was further observed because aged diabetic animals might have become insulin-resistant, and UA plus training intervention has failed to decrease blood glucose levels. 

Also, UA’s neuroprotective effect has been demonstrated by its ability to modulate the Nrf2 pathway in cerebral ischemia in mice. Its ability to improve cognition and anti-oxidant activities in the brains of senescent mice has been reported (35). We used a behavioral paradigm and spatial (open field) and affective (passive avoidance) memory tests to investigate learning and memory impairment. Based on the results, administration of ursolic acid (250 mg/kg) significantly decreased the total distance moved in the open field test but it did not decrease the latency of entrance to the dark compartment in the passive avoidance test, suggesting that administration of ursolic acid could not improve memory function compared with the control group. These findings could suggest that ursolic acid can improve spatial memory and increase learning ability in rats but without change in affective memory. Our study confirms the protective effects of UA (250 mg/kg for eight weeks) against memory impairments in diabetic rats through the open field test; this result is consistent with the findings from a recent study in a T2DM rat model in which UA administration prevented hippocampal cognitive deficiencies (36). 

Oxidative stress plays a fundamental role in the development of diabetic complications and cognitive impairment. The main endogenous defense against oxidative stress involves the Nrf2 dependent anti-oxidant responses, which involve the activation of Nrf2 and the subsequent increased expression of the downstream cytoprotective proteins (37). In the current study, increased oxidative stress indicators were observed in the hippocampus of HFD/STZ-Induced diabetic rats with memory impairments, suggesting more oxidative stress in the diabetic brain. However, there was a parallel increase in the Nrf2 and keap1 nuclear amount, suggesting a relative increment in its related antioxidative responses in the diabetic brain. UA-treated diabetic rats showed an accumulation of nuclear Nrf2 and increased expression of the keap1 protein in the hippocampus, indicating that UA activated a potent Nrf2-mediated adaptive response, which is impaired in diabetes. Accordingly, a number of studies on diabetes have reported that a UA supplement stimulates Nrf2 signaling activation in body’s other regions (22), thus protecting against diabetic complications. Recently, studies showed that ursolic acid might develop the signaling of Nrf2 in hippocampal neurons and cause improvement in memory function (18). However, activating the Nrf2-mediated protective response by consuming ursolic acid might be a potential therapeutic strategy for aged diabetic patients, and further studies to explore the protective effects of UA with Nrf2 activator on diabetes and related mechanisms are required.

UA has also been shown to protect hippocampal neurons against reactive oxygen species and oxidative stress. A study (2004) had similarly reported that UA supplementation could significantly reduce free radical levels in rat neuronal cells (38). Moreover, researchers had found that UA, as a ubiquitous pentacyclic triterpene with an effectively antioxidative activity, could reverse the neurotoxic effect in rat brains by improving the activities of anti-oxidant enzymes, SOD, CAT, GPx, and GR and reducing activated oxygen species content, and in this way boost the memory function in the hippocampal tissues (39). Experimental studies have also shown that UA supplementation could improve the toxic effects of free radicals in middle-aged rats’ hippocampus and boost memory (40, 41). Our study measured the activities of antioxidative enzymes, including CAT, SOD, GPx, and GSH, in the rat brain hippocampus. In the hippocampus of UA-treated rats, there were no significant changes in CAT, GPx, and GSH activities compared with the group control rats, and only a significant effect was observed in training by supplementation interaction effect on SOD. In this study, we investigated ursolic acid effects in the setting of a high-fat diet, likely that is why we did not show significant changes in the anti-oxidant enzymes.

Of note, the hippocampus in older people is very vulnerable due to the reduced capacity of the hemostasis of nerve cells caused by oxidative injuries (42). Researchers have further discovered that the hippocampus controls the aging process (43). Exercise training can thus have beneficial effects on improved brain functioning and quality of life in cases with aged diabetes (44). However, the exact mechanism responsible for the positive effects of exercise training on the brain functioning in such individuals has not yet been well understood. One of the possible mechanisms of the protective ability of exercise training can be the capacity to block the formation of free radicals (15). Study (2009) had further shown that increased levels of anti-oxidant enzymes due to exercise training in different parts of the brain could augment its anti-oxidant capacity (45). Recently, it has been suggested that exercise may prevent synaptic malformations in the hippocampus of diabetic rats (44). Increased exercise also brings beneficial physiological effects in older adults with diabetes, glucose delivery, or IR (46). However, the regulatory effects of endurance exercise and its related mechanism in aged diabetic rats have not yet been fully understood.

There was evidence that exercise could activate Nrf2/keap1 in hippocampal tissue to protect from oxidative stress (15). We observed that there were higher levels of hippocampal Nrf2 and keap1 in trained rats than in control group rats, which suggested that exercise intervention significantly activated Nrf2 and keap1, which was significantly greater in resistance exercise (60% of MVCC), than in endurance exercise (60-75% vVO2max). Endurance exercise raises ADP and AMP which could further activate the energy sensor AMPK, which together with sirtuin-1 (SIRT1), activates alpha-1 activating gamma peroxisome proliferator (PGC-1α). Considering mitochondrial conditions in old age, PGC-1 could also play a prominent role as a key element in mitochondrial biogenesis that increases with endurance training (47). PGC-1α could accordingly interact with other transcription factors such as estrogen-related receptor (ERR), Nrf1, GPx, SOD, CAT, keap1, and Nrf2 to coordinate different programs in response to oxidants (48). This interaction was involved in mitochondrial biogenesis, reducing muscle cell apoptosis and inflammation, which could prevent or delay cell aging (49). Resistance training and other factors such as insulin-like growth factor 1 (IGF-1), insulin, testosterone, and leucine could stimulate the analyzing phosphoinositide 3-kinases PI3K/Akt strain transforming Akt/mTOR signaling pathway (50). The activation of Nrf2 could be further achieved by increasing IGF-1, which enters the brain after exercise training. IGF-1 might thus be a beneficial mediator of exercise training on brain health by activating Nrf2 through increasing Akt and decreasing glycogen synthase kinase 3 beta (GSK-3b) activity (51). 

The above results demonstrate that exercise exerted a cytoprotective effect against diabetes-induced oxidative stress by increasing Nrf2 and keap1 expression. Moreover, trained rats, especially the resistance training group, showed better spatial memory (open field test) than the control group. Evidence suggests that resistance training led to high ROS levels in the hippocampus. In addition, there is a body of evidence linking this type of training with the up-regulation of insulin-like growth factor 1 (IGF-1) and improved cognitive function. Based on the results and our study data, we can hypothesize that resistance training leads to the controlled production of ROS, stimulating IGF-1 signaling. Then, IGF-1 could act as an anti-oxidant protector factor through Nrf2 activity in the hippocampus and improve spatial memory. Several studies have examined the effect of exercise on Nrf2 protein content in brain tissue. Cai *et al*. (2016), who performed an 8-week training study (40 min, 5 days per week), reported a ~13% increase in hippocampal nuclear Nrf2 protein content (15). Some studies have also investigated changes in the brain Nrf2 content after high-intensity resistance training for 12 weeks at 35 cm/sec with a bagged weight attached to animal tails (55-85% 1-RM) and each rat performing 10-12 sets (52, 53). A similar study (2015) also showed an increase in hippocampal Nrf2 protein content consistent with our findings (54). However, a study conducted in 2017 did find that rats that performed a swimming test to exhaustion had a significant decrease in Nrf2 protein content in whole muscle tissue that may be explained by several factors such as exercise modality and intensity and tissue type (55).

However, after exercise intervention, it did not significantly change GPX, CAT, and GSH hippocampal enzyme content, but SOD was markedly increased in trained rats. These changes were caused by exercise. Several studies have examined the effect of exercise on CAT, GPX, GSH, and SOD enzyme content in brain tissue. Researchers showed that regular exercise did not affect GPX and CAT activities in the rats’ brains, which is consistent with our findings (56). Martines *et al*. demonstrated that swimming training improves oxidative stress markers by decreasing lipid peroxidation and increasing the anti-oxidant activity of SOD, CAT, and glutathione peroxidase (GPx)(57). This discrepancy is not apparent, but it might be due to various effects of exercise on the different brain regions or from the disparate methods for assays and exercise methods. 

UA and resistance training increases the expression of hypertrophy-associated genes through improving the insulin-like growth factor 1 (IGF-1) signaling pathway (58). In our study, the effects of concurrent UA and resistance training improved MVCC which probably was due to the activation of hypertrophy pathways and increased strength. Also, the effects of concurrent treatment with UA and endurance training increases vVo2max. Ursolic acid and endurance training both increase AMPK-PGC-1a which plays a key role in mitochondrial biogenesis (59), thereby increasing endurance exercise capacity, which is consistent with a 2018 study (60).

We also considered the effects of resistance/endurance training with UA supplementation on plasma glucose levels, oxidative stress biomarkers, and memory function of the hippocampus in aged Wistar rats with HFD/STZ-induced diabetes. Interestingly, our data demonstrated that exercise and supplement interaction caused no significant change on insulin signaling, memory function, and oxidative stress; only SOD was markedly increased. 

All rats except the control group were fed for four weeks with an HFD formulated with energy from fat (55%), carbohydrates (31%), as well as protein (14%), but the rats in the control group were fed a regular basal diet (10% fat, 75% carbohydrate, and 15% protein). After familiarizing the animals with the laboratory environment (for one week) and the treadmill (for one week), STZ was intraperitoneally (IP) injected (30 mg/kg) in the diabetic groups. Then, all the rats were kept in the laboratory for two weeks (without any interventions). The treatment via endurance/resistance exercise training and UA supplementation also began for eight weeks. After that, the animals were subjected to behavioral studies and sacrificed for histological and molecular studies

DU: Values expressed as the Mean±SEM. Data were analyzed by repeated measures, n=8 per group

U: diabetic+ursolic acid; DR: diabetic+resistance exercise training; DRU: diabetic+resistance exercise training+ursolic acid; DE: diabetic+endurance exercise training; DEU: diabetic+endurance exercise training+ursolic acid; D: diabetic; C: control

Values are expressed as the Mean±SEM. Data were analyzed by repeated measures, n=8 per group

U: diabetic+ursolic acid; DR: diabetic+resistance exercise training; DRU: diabetic+resistance exercise training+ursolic acid; DE: diabetic+endurance exercise training; DEU: diabetic+endurance exercise training+ursolic acid; D: diabetic; C: control

Values are expressed as the Mean±SEM. Data were analyzed by one-way ANOVA, n=8 per group. ** *P≤*0.01 compared the DR and D to C between groups. € *P≤*0.05 compared the DU to C between group

U: diabetic+ursolic acid; DR: diabetic+resistance exercise training; DRU: diabetic+resistance exercise training+ursolic acid; DE: diabetic+endurance exercise training; DEU: diabetic+endurance exercise training+ursolic acid; D: diabetic; C: control

Values are expressed as the Mean±SEM. Data were analyzed by one-way ANOVA, n=8 per group. *** *P≤*0.001 compared the DR to DE, DEU, DU, and D between groups. $$ *P≤*0.01 compared the DEU to C between groups

U: diabetic+ursolic acid; DR: diabetic+resistance exercise training; DRU: diabetic+resistance exercise training+ursolic acid; DE: diabetic+endurance exercise training; DEU: diabetic+endurance exercise training+ursolic acid; D: diabetic; C: control

Values are expressed as the Mean±SEM. Data were analyzed by one-way ANOVA, n=8 per group. £ *P≤*0.05 compared the D to DRU and DEU between groups.

U: diabetic+ursolic acid; DR: diabetic+resistance exercise training; DRU: diabetic+resistance exercise training+ursolic acid; DE: diabetic+endurance exercise training; DEU: diabetic+endurance exercise training+ursolic acid; D: diabetic; C: control

Values are expressed as the Mean±SEM. Data were analyzed by one-way ANOVA, n=8 per group. *** *P≤*0.001 compared the DU to DEU and DE between groups. $$ *P≤*0.015 compared the DU to DR between groups

U: diabetic+ursolic acid; DR: diabetic+resistance exercise training; DRU: diabetic+resistance exercise training+ursolic acid; DE: diabetic+endurance exercise training; DEU: diabetic+endurance exercise training+ursolic acid; D: diabetic; C: control

Values are expressed as the Mean±SEM. Data were analyzed by one-way ANOVA, n=8 per group

U: diabetic+ursolic acid; DR: diabetic+resistance exercise training; DRU: diabetic+resistance exercise training+ursolic acid; DE: diabetic+endurance exercise training; DEU: diabetic+endurance exercise training+ursolic acid; D: diabetic; C: control

## Conclusion

As the first attempt, this study investigated the effects of resistance or endurance training associated with UA supplementation on Nrf2/Keap1/ARE signaling and anti-oxidant defense pathways in HFD/STZ-induced diabetic aged male Wistar rats. Despite some improvements in blood glucose, MVCC, anti-oxidant markers, and Nrf2, the results demonstrated no significant effects on cognitive-motor dysfunction tests. Thus, further studies are needed to develop novel practical nutritional strategies involving UA supplementation to clarify how chronic UA supplementation with exercise training can be recruited to treat hippocampal complications and cognitive-motor dysfunction caused by diabetes.

## Authors’ Contributions

 S A, M F, E B, and E S devised the research. S A, M F, and E B were the supervisors of the training protocols and laboratory exams, as well as data collection. S A and M F addressed data analysis and interpretation. S A, M F, E B, and E S compiled the first draft of this manuscript. M F was responsible for editing the manuscript. Each author contributed to the manuscript writing, they also read and confirmed the final draft.

## Data Availability statement

The data used to support the findings of this study are available from the corresponding author upon request.

## Conflicts of Interest

There were no conflicts interest.
